# Can We Reduce the Duration of Untreated Psychosis? A Systematic Review and Meta-Analysis of Controlled Interventional Studies

**DOI:** 10.1093/schbul/sbx166

**Published:** 2018-01-24

**Authors:** Dominic Oliver, Cathy Davies, Georgia Crossland, Steffiany Lim, George Gifford, Philip McGuire, Paolo Fusar-Poli

**Affiliations:** 1 Early Psychosis: Interventions & Clinical-Detection (EPIC) Lab, Department of Psychosis Studies, Institute of Psychiatry, Psychology and Neuroscience, King’s College London, UK; 2 Department of Psychosis Studies, IoPPN, King’s College London, UK; 3 OASIS Service, South London and the Maudsley NHS National Health Service Foundation Trust, UK; 4 National Institute for Health Research (NIHR) Biomedical Research Centre for Mental Health, IoPPN, King’s College London, UK

**Keywords:** duration of untreated psychosis, schizophrenia, first episode, prevention, outcomes, meta-analysis

## Abstract

Reduction of duration of untreated psychosis (DUP) is the key strategy of early interventions for improving the outcomes of first-episode psychosis. Although several controlled interventional studies have been conducted with the aim of reducing DUP, the results are highly inconsistent and conflicting. The current study systematically searches Web of Science and Ovid for English original articles investigating interventions adopted to reduce DUP, compared to a control intervention, up to April 6, 2017. Sixteen controlled interventional studies were retrieved, including 1964 patients in the intervention arm and 1358 in the control arm. The controlled intervention studies were characterized by standalone first episode psychosis services, standalone clinical high risk services, community interventions, healthcare professional training, and multifocus interventions. Random effects meta-analyses were conducted. There was no summary evidence that available interventions are successful in reducing DUP during the first episode of psychosis (Hedges’ *g* = −0.12, 95% CI = −0.25 to 0.01). Subgroup analyses showed no differences within each subgroup, with the exception of clinical high risk services (Hedges’ *g* = −0.386, 95% CI = −0.726 to −0.045). These negative findings may reflect a parceled research base in the area, lack of prospective randomized controlled trials (only 2 randomized cluster designed studies were present) and small sample sizes. There was substantial heterogeneity (*I*^2^ = 66.4%), most of which was accounted by different definitions of DUP onset (*R*^2^ = .88). Psychometric standardization of DUP definition, improvement of study design, and implementation of preventative strategies seem the most promising avenues for reducing DUP and improving outcomes of first-episode psychosis.

## Introduction

Psychosis is one of the most debilitating psychiatric conditions with limited options to improve outcomes.^1^ One key strategy is reducing the duration of untreated psychosis (DUP), the time from the first symptom of psychosis to the start of treatment.^2^ Accumulating studies have shown that a longer DUP is associated with poorer outcomes for people with first-episode psychosis (FEP), including worse positive^3,4^ and negative symptom^4,5^ severity, poorer rates of remission,^4,6,7^ poorer social cognition,^6,8^ and cognitive impairment.^6,9,10^ In addition to the clinical, functional, and cognitive benefits, reducing DUP is associated with reducing the social consequences of psychosis onset, such as social isolation, unemployment, homelessness, and can reduce deliberate self harm^11,12^ and violence toward others.^12,13^ Under standard care, DUP tends to be quite long, with means varying between 22 weeks and over 150 weeks,^14^ with high heterogeneity between patients.^15^ These long periods without treatment arise from several sources, both intrinsic (eg, symptom severity, patients’ attitude) and extrinsic (eg, access to care).^16^ These considerations lay foundations for specialized early intervention services^1^ with the aim of improving early detection and facilitating pathways to care and treatment; minimizing DUP.

In recent years, many different interventions have been trialed to reduce DUP, targeting different sources of referrals for FEP cases such as general practitioners, service providers, and the community. These specific interventions have been varied including programs networking primary healthcare providers and public education,^17^ early detection programs identifying FEP patients^12,18^ or those at clinical high risk for psychosis (CHR-P),^19,20^ information workshops,^21^ written information in information packs, newsletters or brochures,^17,22^ community intervention teams and activities,^18^ and websites and telephone hotlines.^17^ Studies have typically compared service providers (standalone FEP services or standalone CHR-P services), community, healthcare professional training or multifocus (combining other types) interventions to a control group. The overall impact of these controlled interventions for reducing the DUP is still undetermined because the findings are conflicting.^23,24^ Earlier reviews suggested that interventions reducing DUP held promise for producing better outcomes in FEP,^16,23^ but these studies did not combine qualitative and quantitative data synthesis. Furthermore, in the years since the last review,^16^ there have been significant additions to the literature.^20,25–30^

The primary aim of the current study is to systematically review the conflicting evidence for the impact of specific controlled interventions in reducing DUP and to provide a meta-analysis measuring their magnitude, consistency, determining which type of intervention is the most successful, as well as addressing potential confounders. These analyses may be particularly informative for the implementation of early psychosis services worldwide.

## Methods

### Selection Procedures and Data Collection

#### Search Strategies.

PRISMA^31^ and MOOSE guidelines^32^ were adhered to throughout to achieve high quality of reporting (supplementary eTables 1 and 2). Details of the protocol for systematic review were registered on PROSPERO (CRD42017057082).

A 2-step systematic search of the literature was performed by 2 independent researchers (S.L. and G.C.) to identify relevant studies investigating the effect of controlled interventions aiming to reduce DUP in early psychosis.^16^

At a first step, the Web of Science database by Thomson Reuters (including Web of Science, BIOSIS Citation Index and MEDLINE) and the Ovid database by Wolters Kluwer (including MEDLINE and PsycINFO) were searched. Keywords used were (“duration of untreated psychosis”) AND (intervention OR decreas* or reduc*). The search was extended from inception until April 6, 2017.

The second step involved an electronic manual search of references found in the included articles. Articles found through these steps were then screened on the basis of title or abstract reading. Articles that remained were then fully downloaded as PDFs and assessed for eligibility on the basis of full text reading. Disagreements regarding studies fitting inclusion criteria were resolved by consensus with a third researcher (D.O.).

#### Inclusion Criteria.

Articles meeting the inclusion criteria for the current systematic review and meta-analysis: (*a*) were original articles or original data presented as conference abstract, written in English, (*b*) included patients diagnosed with a psychotic disorder defined according to standard international Diagnostic and Statistical Manual of Mental Disorders (DSM) or International Statistical Classification of Diseases and Related Health Problems (ICD) criteria—any version, (*c*) were controlled studies with either randomized cluster, cohort, or cohort analytic designs, and (*d*) reported DUP as a key outcome measure, as defined by each individual article. We did not restrict inclusion to any specific study design.

#### Exclusion Criteria.

We excluded (*a*) pilot datasets, reviews, articles in languages other than English, (*b*) articles failing to report enough data to perform a meta-analysis (authors were contacted to obtain missing data), and (*c*) studies with overlapping cohorts (only the study with the largest sample or most recently published was included).

#### Recorded Variables.

We recorded the following variables from each article relating to patients: mean age, gender, ethnicity (% white), marital status (proportion of married subjects), cannabis abuse/dependence (% meeting abuse/dependence criteria), alcohol abuse/dependence (% meeting abuse/dependence criteria), living status (% living independently), migrant status (% nonmigrants), psychotic symptom severity at baseline (PANSS), diagnosis (% schizophrenia); relating to the study: author, length of intervention (months) (time intervention was delivered to community, healthcare professionals, etc.), quality of studies (see below), publication year, continent, healthcare system type (Semashko, Bismarck, market-based), type of controlled interventions (standalone FEP services, standalone CHR-P services, community interventions, healthcare professional training, and multifocus interventions), primary media of intervention (written or audiovisual information, direct contact or service configuration, defined below), study design (randomized cluster, cohort analytic, cohort), target of intervention, control DUP, definition of DUP onset, definition of DUP endpoint.

The main outcome was DUP in intervention groups and control groups. We extracted meta-analytical DUP data (see “Statistical Analysis” section) and DUP definitions (at onset and endpoint).

#### Quality Assessment.

Evaluation of quality of studies was performed using a risk bias tool, an adapted version of the Newcastle Ottawa Scale^33^ (see supplementary eMethods 1).

### Statistical Analysis

The effect-size measure was Hedges’ *g*.^34^ This indexed the impact of the specific intervention on the DUP. Negative values indicated reduced DUP in the intervention group and positive values indicated reduced DUP in the control group. The DUP was primarily measured through mean value (in days) and standard deviation (SD) or median value (in days) and standard error of the mean (SEM) (when presented instead of mean and SD) and group size (*n*). Where these values were not available other complementary statistics allowing the estimation of Hedges’ *g* were extracted.

Controlled interventional strategies were defined as: (*a*) standalone FEP services, (*b*) standalone CHR-P services, (*c*) community interventions, (*d*) healthcare professional training, and (*e*) multifocus interventions. These subgroups were defined by the systematic review that was conducted ahead of the meta-analysis, as indicated below. The meta-analyses included an overall estimate across all subgroups as well as within-subgroup summary effects and between-subgroups effects.

Heterogeneity among study point estimates was assessed using *Q* statistics^35^ and the proportion of the total variability in the effect size estimates evaluated with the *I*^2^ index.^35^ Given the methodological heterogeneity of the included studies, random effects models^36^ were employed using the method of DerSimonian and Laird.^37^

Risk of publication bias was tested by visual inspection of funnel plots in addition to the application of the Egger regression intercept method^38^ and the Duval and Tweedie “trim and fill” method.^39^ To further assess the robustness of the results, we performed sensitivity analyses by sequentially removing each study and re-running the analysis.^40^

To explain any heterogeneity found, meta-regression analyses were conducted when at least 10 studies were available for the specific confounders relating to patients: mean age, gender, ethnicity (% white), marital status (proportion of married subjects), cannabis abuse/dependence (% meeting abuse/dependence criteria), alcohol abuse/dependence (% meeting abuse/dependence criteria), living status (% living independently), migrant status (% non-migrants), psychotic symptom severity at baseline (PANSS), diagnosis (% schizophrenia); relating to the study: length of intervention (months), quality of studies (see below), publication year, continent, healthcare system type (Semashko, Bismarck, market-based), study design (randomized cluster, cohort analytic, cohort), control DUP, definition of DUP onset, definition of DUP endpoint. When PANSS scores were not available for symptom severity, SAPS/SANS or BPRS scores were converted to PANSS, following previously established procedures.^41,42^

The significance level was set to 0.05 (2-sided). Meta-analyses were performed using Comprehensive Meta-Analysis Software version 3^43^ and STATA version 13.^44^

## Results

### Database

**Fig. 1.**
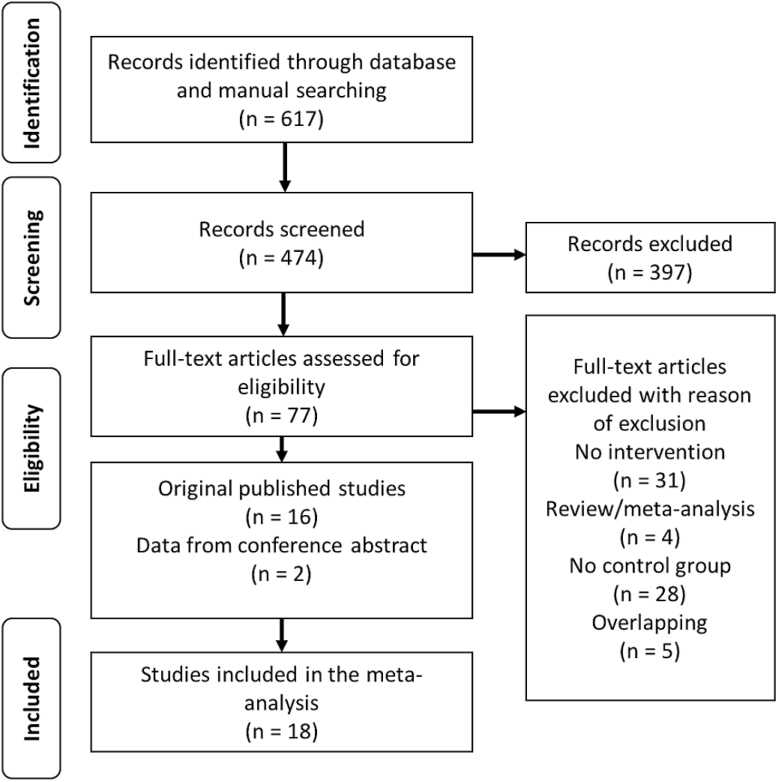
PRISMA flow chart.

The flow of articles through the initial literature search, including numbers of articles screened, assessed for eligibility and included in the review, is summarized in the PRISMA plot (figure 1). The search uncovered 14 independent articles (7 new^20,25–30^ compared to the previous systematic search^16^) in addition to 2 new conference abstracts.^45,46^ Five of the previously found studies were excluded, one for not including a control group,^47^ another for not explicitly reporting DUP^22^ and 3 for overlapping data.^23,48,49^ The final database comprised 16 studies including 1964 patients in the intervention arm and 1358 in the control arm. All studies used similar definitions of FEP, with 12 studies defining it according to DSM-IV,^15,20,21,24–29,45,46,50^ 2 according to DSM-III-R,^17,18^ 1 according to ICD-10,^51^ and 1 employing both ICD-10 and DSM-IV^30^. The mean age of the intervention and control groups were 25.5 years (range 21.6–36.6) and 26.4 years (range 22.0–38.0), respectively. The mean length of intervention was 31.3 months (range 8–78 months). All the studies had either cohort,^17,18,24–27,29,30,45,50^ cohort analytic,^15,20,28,46^ or randomized cluster design.^21,51^ More information on interventions is detailed in table 1.

**Table 1.** Description of Included Studies

Initiative	Location (Health care Provision Type)	Duration of Intervention	Intervention, *n*	Control, *n*	Age (SD)	DUP Definition	FEP Definition	Intervention Type (Target)
CIEIS^29^	London, UK (Semashko)	12 months	104	66	22.4 (6.3)	Retrospective, clinical	DSM-IV	Community intervention (Community workers— non-healthcare)
DETECT^25^	Dublin, Ireland (Bismarck)	78 months	172	151	26.2 (IQR: 20.9, 36.0)	Retrospective, clinical	DSM-IV	Multifocus intervention (GPs, general public, universities)
EASY/JCEP^26^	Hong Kong (Semashko)	12 months	479	122	31.6 (8.4)	Retrospective, clinical	DSM-IV	Community intervention (general public)
EDEN^28^	Birmingham, UK (Semashko)	18 months	77	74	30.9	Retrospective, clinical	DSM-IV	Community intervention (general public, patients)
EPIP^17^	Singapore (Bismarck)	2 years	287	107	25.4 (8.5)	Retrospective, clinical	DSM-III-R	Multifocus intervention (GPs, general public, patients, patients’ families)
EPPIC^18^	Melbourne, Australia (Bismarck)	8 months	51	51	22.4 (3.8)	Retrospective, clinical	DSM-III-R	Standalone FEP service
EPPIC^24^	Melbourne, Australia (Bismarck)	12 months	40	58	22.2 (3.4)	Retrospective, clinical	DSM-IV	Multifocus intervention (GPs, secondary schools)
IMAGES^30^	Jujuy, Argentina (Bismarck)	6 years	53	53	30.7 (11.1)	Retrospective, clinical	DSM-IV/ ICD-10	Healthcare professional training (health workers)
LEOCAT^21^	London, UK (Semashko)	27 months	36	35	23.9 (5.27)	Retrospective, psychometric	DSM-IV	Healthcare professional training (GPs)
OASIS^20^	London, UK (Semashko)	5 years	43	147	24.0 (5.5)	Prospective, psychometric	DSM-IV	Standalone ARMS service
PAE-TPI^46^	Barcelona, Spain (Semashko)	15 months	133	58	-	Retrospective, clinical	DSM-IV	Standalone FEP service
PEPP^50^	Ontario, Canada (Semashko)	2 years	85	84	25.0 (7.8)	Retrospective, clinical	DSM-IV	Multifocus intervention (GPs, general public, high school students, university students, patients’ families)
PEPP^27^	Ontario, Canada (Semashko)	42 months	145	132	24.3 (4.0)	Retrospective, clinical	DSM-IV	Healthcare professional training (GPs)
REDIRECT^51^	Birmingham, UK (Semashko)	30 months	65	58	22.7 (3.7)	Retrospective, clinical	ICD-10	Healthcare professional training (GPs)
STEP^45^	USA (market-based)	18 months	53	22	—	Retrospective, psychometric	DSM-IV	Multifocus intervention (secondary schools, GPs, acute clinical care providers, community agencies)
TIPS^15^	Rogaland County, Norway (Bismarck)	4 years	141	140	28.6 (9.1)	Retrospective, psychometric	DSM-IV	Multifocus intervention (GPs, general public, healthcare workers)

*Note:* FEP, first-episode psychosis; DUP, duration of untreated psychosis; DSM, diagnostic and statistical manual of mental disorders; ICD, International Statistical Classification of Diseases and Related Health Problems.

### Systematic Review of the Type of Interventions for Reducing the DUP

All included studies were investigating an intervention aiming to reduce DUP compared to a control group. DUP onset was operationalized as patient report of onset of frank psychotic general symptoms in 8 studies,^18,24,25,27,29,30,50,51^ as patient report of onset of frank psychotic positive symptoms in 4 studies,^17,20,26,45^ defined psychometrically using PANSS in 2 studies^15,28^ or unremitting psychotic symptoms for greater than 1 week in 1 study.^21^ DUP endpoint was operationalized as general commencement of antipsychotic treatment in 12 studies,^15,17,18,20,21,24–26,28,30,45,51^ 30 days’ consistent antipsychotic medication in 2 studies^27,50^ or first contact with FEP services in 1 study.^29^ Information on DUP onset and endpoint definition used was missing in one study.^46^ All but one^20^ defined the DUP retrospectively.^15,17,18,21,24–30,45,46,50,51^ Most studies were performed in high-income countries on urban populations,^15,17,18,20,21,24–29,45,46,50,51^ with the only exception focusing on a rural population in Argentina.^30^ Three studies^18,20,46^ focused on service configuration alone as main type of intervention. Two of these investigated the effect of FEP services^18,46^ with the other focusing on the effect of CHR-P services.^20^ The 2 approaches were quite distinct in that the CHR-P service was the only one to adopt a longitudinal design. The remaining studies combined early intervention with education-based interventions. Specifically, 3 studies^26,28,29^ focused entirely on community interventions: psychosis awareness campaigns using promotional material for early intervention services, talks, and exhibitions aimed at the public. Seventy-five percent of community intervention studies combined direct contact with audiovisual promotional materials. A further 4 studies^21,27,30,51^ targeted healthcare professionals. All studies emphasized direct contact, particularly through workshops but only half incorporated audiovisual materials. A further 6 studies were multifocus interventions, combining different types of service configuration with community education campaigns and healthcare professional training.^15,17,24,25,45,50^ It was thus possible to cluster the types of controlled interventions across 5 subgroups: standalone FEP services, standalone CHR-P services, community interventions, healthcare professional training, and multifocus interventions (more detail on the content of each intervention can be seen in supplementary eTable 3).

### Meta-Analytical Summary of Interventions for Reducing DUP

**Fig. 2.**
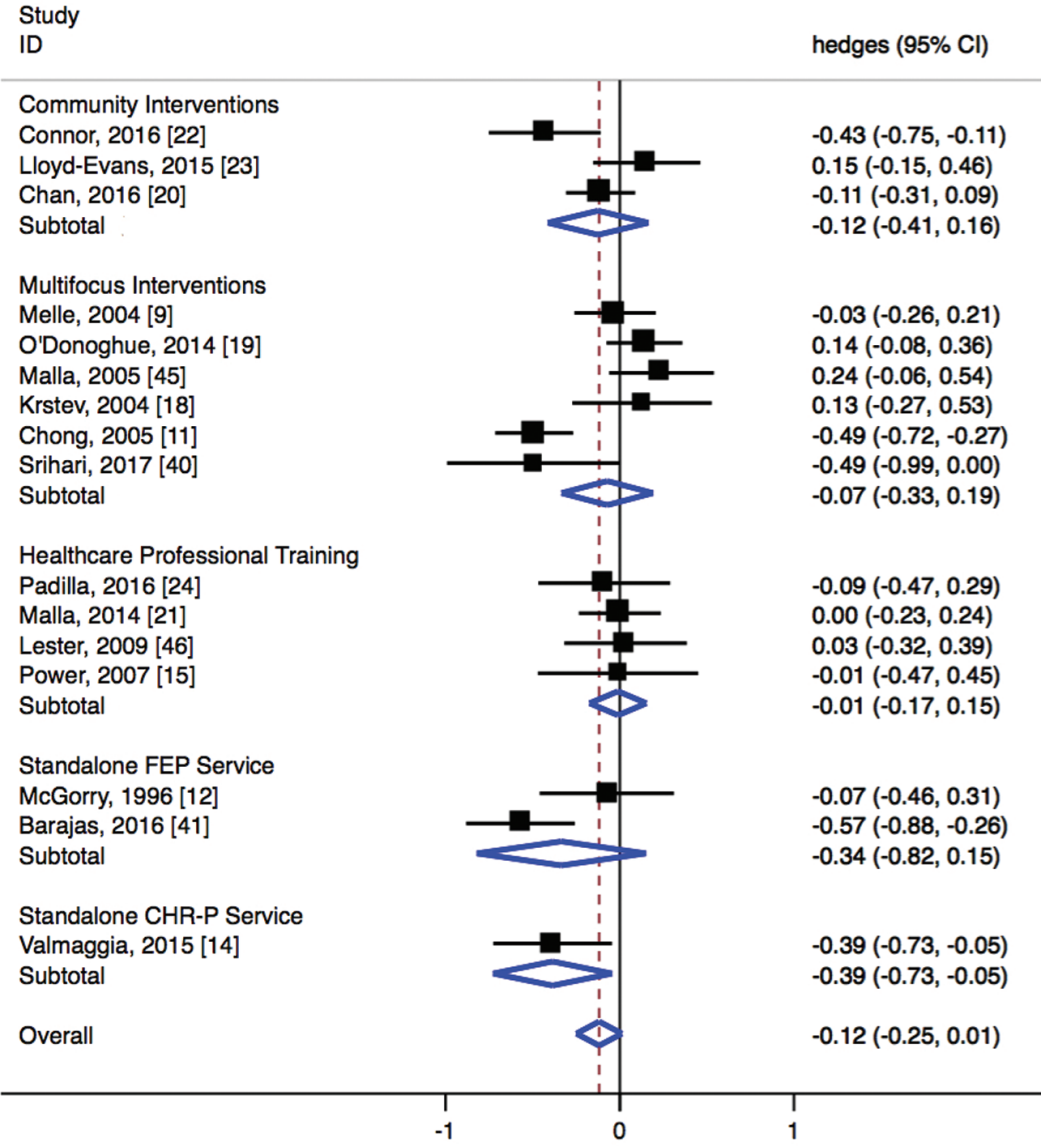
Forest plot showing results of random effects within-subgroup analysis and overall summary effect.

The overall effect for DUP reduction across all the included studies was small (Hedges’ *g* = −0.12, 95% CI = −0.25 to 0.01) and did not reach significance (*P* = .077; figure 2). When only including studies published in peer-reviewed journals, there were no significant changes (Hedges’ *g* = −0.07, 95% CI = −0.20 to 0.05).

### Subgroups Meta-Analysis of Interventions for Reducing DUP

Within-subgroup analysis showed no significant effects within 4 of the 5 subgroups (figure 2): multifocus interventions (Hedges’ *g* = −0.014; 95% CI = −0.291 to 0.263), community interventions (Hedges’ *g* = −0.186; 95% CI = −0.451 to 0.079), healthcare professional training (Hedges’ *g* = −0.010, 95% CI = −0.173 to 0.152) and standalone FEP service (Hedges’ *g* = −0.366, 95% CI = −0.821 to 0.150). Conversely, standalone CHR-P services did significantly reduce DUP (Hedges’ *g* = −0.386, 95% CI = −0.726 to −0.045). However, this was very underpowered with only one study. Furthermore, between-subgroup analyses showed no significant differences across the 5 types of intervention (*Q* = 9.283, *df* = 4, *P* = .054).

### Heterogeneity, Meta-Regressions, Publication Bias, and Sensitivity Analysis

There was substantial heterogeneity present in the meta-analysis, with an *I*^2^ of 66.4% (*Q* = 44.697, *P* < .001, *df* = 15), which justified conducting meta-regression analyses.

**Fig. 3.**
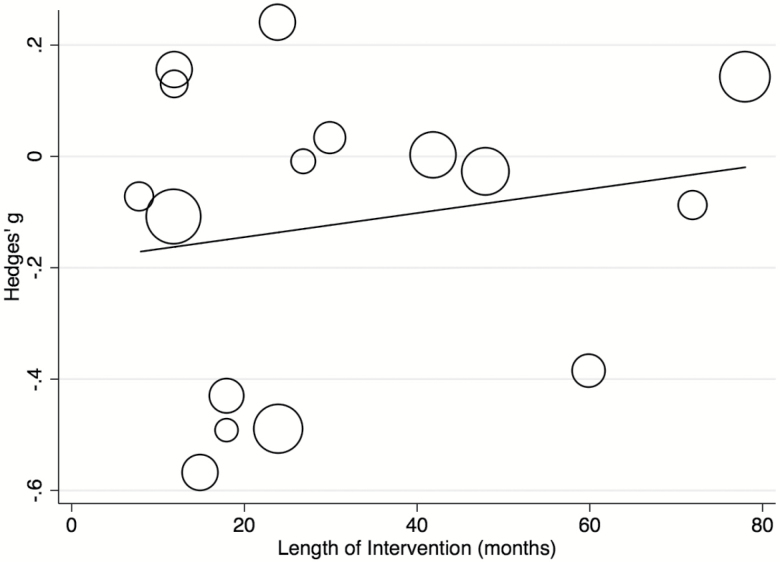
Meta-regression analyses. Effect of controlled intervention on the DUP (Hedges’ g) by length of intervention (months).

**Fig. 4.**
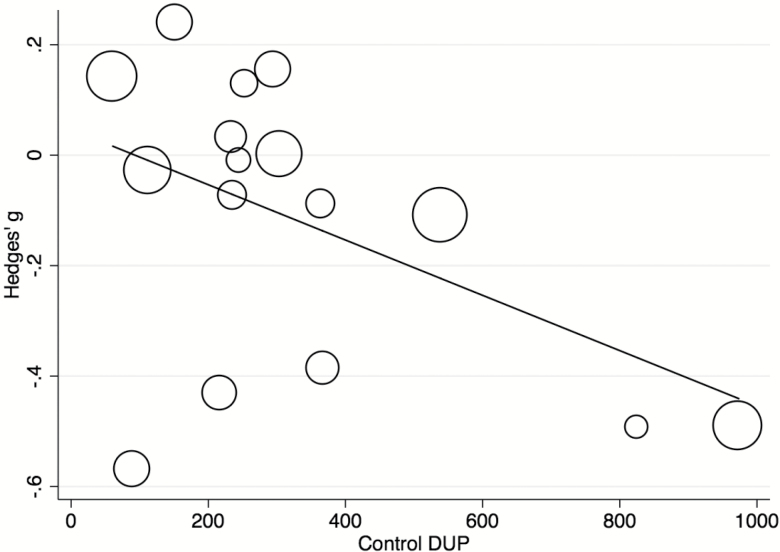
Meta-regression analyses. Effect of controlled intervention on the DUP (Hedges’ g) by control DUP (days).

**Fig. 5.**
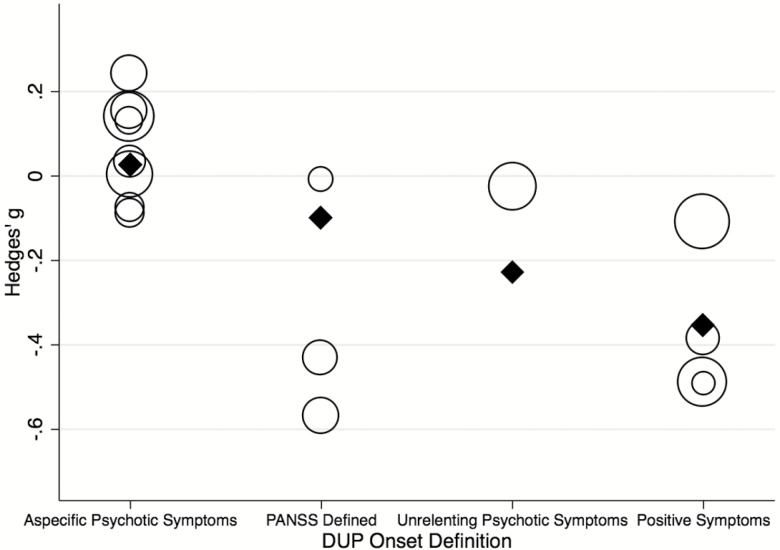
Meta-regression analyses. Effect of controlled intervention on the DUP (Hedges’ g) by DUP onset definition.

Meta-regressions were conducted on (1) complete set of studies for length of intervention, publication year, study design and control DUP, (2) *n* = 15 for quality of studies, definition of DUP onset and endpoint, (3) *n* = 14 for mean age gender, and (4) *n* = 10 for marital status. Analyses for other factors mentioned in the methods were not performed as fewer than 10 studies contributed relevant data. There were no significant effects for the following patient-related variables: age (β = −0.022, *P* = .274), gender (β ≤ 0.001, *P* = .973), marital status (β = 0.006, *P* = .708), or for the following study-related variables: length of interventions (β = 0.002, *P* = .473; figure 3), quality of studies (β = 0.070, *P* = .396), publication year (β = −0.012, *P* = .317), continent (*Q* = 1.73, *P* = .786), healthcare system type (*Q* = 1.36, *P* = .507), study design (*Q* = 4.13, *P* = .127) or definition of DUP endpoint (*Q* = 1.15, *P* = .562) see supplementary eFigures. Meta-regression of control DUP showed that a longer DUP at the start of the study was associated with a greater reduction in DUP (β < −0.001, *P* = .028). This model explained over a third of the total between-study variance (*R*^2^ = .37; figure 4). However, this effect was mostly driven by 2 outliers^17,45^; when these were removed, the effect was no longer significant (β < −0.001, *P* = .115). Meta-regression of definition of DUP onset showed that defining the DUP onset as the onset of frank psychotic positive symptoms or by using the PANSS was associated with a significantly greater decrease in DUP compared to other onset definitions (β = −0.400, *P* < .001 and β = −0.480, *P* < .001, respectively) (figure 5). This model explained a substantial proportion of total between-study variance (*R*^2^ = .88). A multivariate meta-regression model including control DUP and definition of DUP onset was tested but was nonsignificant (*P* = .0536, *R*^2^ = .39).

There was no evidence of publication bias as indicated by visual inspection of the funnel plots (supplementary eFigures 10 and 11) and by the Egger regression intercept (intercept = −0.508, *P* = .386). Sensitivity analysis did not suggest sensitivity of the meta-analytic estimate to the removal of any one study (supplementary eFigure 12), confirming the robustness of the findings.

## Discussion

This is the first meta-analysis of the impact of controlled interventional studies to shorten DUP in FEP. We found 16 controlled studies investigating interventions to reduce DUP in FEP patients. The database was relatively large with 1964 patients in intervention groups and 1358 in control groups with duration of interventions lasting an average of 31.3 months. There was no overall evidence that they were effective in reducing the DUP. Within-subgroup analyses did not provide evidence that standalone FEP services, community interventions, healthcare professional training, or multifocus interventions are effective in reducing DUP compared to control. Although there was some evidence that standalone CHR-P services are effective in reducing DUP compared to control, this analysis had low power and between-subgroup analysis showed no superiority of any type of intervention. There were no publication biases and the results were not affected by outliers. The heterogeneous definition of DUP onset explained a substantial proportion of the observed variance.

The principal finding of this meta-analysis is lack of overall summary evidence for any beneficial impact of controlled interventions for reducing the DUP. Our finding corroborates earlier systematic reviews concluding that the evidence base for effective reductions of DUP was “very limited.”^16^ Negative findings are always difficult to interpret because “absence of evidence is not equivalent to evidence of absence.”^52^ However, ours are unlikely to be secondary to low power since the analyses are based on a larger sample compared to previous systematic reviews^16^ and the overall effect was very small in magnitude. Furthermore, the negative findings were robust and not due to publication biases or to the presence of outliers. Moreover, when the analyses were stratified within and between the different subgroups the results were overall unchanged. Within subgroup analyses indicated that the lack of DUP reduction was specific to standalone FEP services, community, healthcare professionals training or multifocus interventions. The only exception was noted in relation to standalone CHR-P services. However, this is the only report available; these findings should be interpreted cautiously. Furthermore, the magnitude of the effect size for this study was still small to medium and between-subgroup analysis highlighted no significant effect of subgroup on DUP.

Shortening DUP is influenced by many different factors. It is possible that the negative findings may be the consequence of substantial heterogeneity both at a patient-level and across individual studies. Variation in DUP values within each study was high and this raises the question of individuality of cases and the factors that will vary from patient to patient. We attempted to address study-level heterogeneity with meta-regression analyses on numerous patient related variables (mean age, gender, marital status) and study related variables (length of intervention, quality of studies, publication year, continent, study design, and definitions of DUP at endpoint), that revealed no significant effects. We found that a shorter start DUP (control DUP) leaves less opportunity for reducing DUP, however, this effect was very small and influenced by outliers. Conversely, the definition of DUP onset had a stronger impact on reducing DUP, explaining more than half of the observed heterogeneity. The definition of DUP is not precisely operationalized and still subject to variable ascertainment.^16,53^ Defining onset psychometrically using the PANSS or with positive symptoms using a clinical instrument was linked with a significant reduction in DUP while other definitions were not (figure 5). This suggests the usefulness of psychometric and clinical instruments as a way of reliably defining the onset of DUP. Proper psychometric studies are however required to standardize DUP definition. For example, issues have previously been raised regarding the pseudospecificity of the PANSS (eg, correlations between different subdomains that are not entirely independent).^54^

The current meta-analysis has some potential clinical implications. It highlights that, although the positives for reducing DUP seem obvious, accomplishing this is difficult. This meta-analysis has not provided convincing evidence that current approaches to reduce DUP are effective in accomplishing their aim, with the potential exception of CHR-P services. Our findings are particularly concerning if interpreted along with the other recent meta-analytical negative findings showing that specialized early intervention programs based on integrated psychopharmacological, psychoeducational, and psychological interventions are not more effective in preventing a relapse following a FEP than standard care.^1^ From a purely meta-analytical perspective, these 2 recent meta-analyses indicate that despite all efforts, there is not yet convincing, robust evidence that early interventions in psychosis can reliably reduce DUP or prevent relapse, even when cutting edge treatments are implemented under well-resourced research scenarios. Although such a gloomy figure may shed pessimistic light on the field of early interventions in psychosis, our findings should be interpreted cautiously. Meta-analyses inherit the methodological limitations of the underlying individual studies. It is possible that small sample size (over half of included studies had *n* < 100 in intervention or control group), suboptimal study designs and idiosyncratic interventions do not allow retrieval of consistent effects. Indeed, we found that the definition of the DUP was unstandardized and associated with significant heterogeneity. Thus, it seems clear that some standardization of DUP intervention studies should be on the research agenda for the near future. Similarly, differences in how a “first” episode is defined could further add heterogeneity, potentially referring to first contact with services for a psychotic disorder, first adequate treatment for a psychotic disorder or presenting within a specific amount of time since symptom onset.^55^ Furthermore, since there are many differences between interventions, even within subgroups, standardization would greatly benefit future comparability analyses. While 2 studies employed randomized cluster designs,^21,51^ no randomized controlled trials (RCTs) were found in the search, which is unsurprising given the difficulties in performing them. Randomization at the patient level is logistically complex, randomized clustering is more viable as randomization is performed at the practice level. Moreover, the ethics of withholding interventions with no discernible adverse effects from FEP patients is complicated, particularly those relating to the critical loss period^56^: the intervention only has benefit before endpoint of treatment, so the patient can derive no benefit after study completion. Despite the difficulties in implementing randomized clustering trials into the field, it is important at least to improve the consistency and comparability between studies. These issues can only be tackled by large-scale collaborations across research consortia. Overall, there is a clear implementation gap to be filled for extending the benefits of early interventions in psychosis.^57^ However, meta-regression on study design showed no significant reduction in DUP when random clustering was used compared to other designs, though power was low.

A final clinical consideration relates to the potential role of psychosis prevention. As highlighted by our within subgroup analysis, the CHR-P approach has unique potential for altering the DUP and therefore the course of psychosis.^58^ Controlled interventions in CHR-P services can be highly effective as they can tackle some of the limitations above. The study investigating the effect of CHR-P services^20^ is unique as it adopts a prospective, longitudinal design. Consequently, the accuracy of DUP definition is the highest in this scenario and was operationalized with standardized psychometric instruments such as the Comprehensive Assessment of At Risk Mental State (CAARMS, version 12/2006), which accurately estimate timing of onset of attenuated and frank psychotic symptoms prospectively.^59^ Future controlled randomized interventional studies could use similar psychometric instruments to define the DUP. Importantly, in this study, the average DUP for those who had contacted CHR-P services was 11.2 days compared to 366.5 in FEP services.^20^ Beyond DUP reduction, the CHR-P services further allow for primary indicated prevention, halving the risk of progression to psychosis.^60^ This may be particularly important,^57^ given the lack of robust effects on DUP reduction at the FEP stage. A third benefit of CHR-P services may extend to those already identified as FEP at the time of the initial CHR-P assessment. In fact, compared to patients accessing FEP services, patients who presented in the CHR-P stage are also less likely to require admission following the onset of psychosis (46% vs 68%) and less likely to require a compulsory admission in the short-term (30% vs 62%).^61^ However, the implementation of indicated prevention strategies in mental healthcare is still limited and not widely accessible^57,62^ and, moreover, there is recent evidence indicating that only about half of first episode patients have experienced CHR-P symptoms before illness onset.^63^ Furthermore, new interventional studies in children and adolescents are needed to clarify potential benefits of DUP reduction in earlier stages.

However, beyond their effectiveness on DUP reduction, early psychosis services have been crucial in changing how mental health and mental healthcare are viewed worldwide.^53^ Even if not (yet) robustly justified by meta-analytical data, such initiatives provide humane, trust-engendering support to patients and families at a difficult moment in their lives.^64^

## Conclusion

This meta-analysis provides a significant quantitative summary of current evidence for interventions to reduce DUP during FEP. It shows a lack of robust evidence that specific interventions such as standalone FEP services, community interventions, healthcare professional training, and multifocus interventions are successful in accomplishing this. While this finding is negative, there is some evidence that initiatives to reduce DUP may be effective in areas where the DUP is particularly long. Some evidence is also emerging for CHR-P services but it is not replicated. Collaborative large-scale initiatives adopting well standardized psychometric definitions of DUP and refined study designs are needed to advance knowledge and improve outcomes of FEP.

## Supplementary Material

Supplementary data are available at *Schizophrenia Bulletin* online.

Supplementary_Material
