# Supplementary material for: Can We Reduce the Duration of Untreated Psychosis? A Systematic Review and Meta-Analysis of Controlled Interventional Studies
Source: Schizophr Bull. 2018 Jan 24;44(6):1362–72. doi: 10.1093/schbul/sbx166 (PMC6192469; doi:10.1093/schbul/sbx166)
Supplement: Supplementary_Material [file sbx166_suppl_supplementary_material.doc]

| **eTable 1:** MOOSE guidelines for meta-analysis and systematic reviews | | **Reported on page #** |
| --- | --- | --- |
| **Title** | Identify the study as a meta-analysis (or systematic review) | **1** |
| **Abstract** | Use the journal’s structured format | **2** |
| **Introduction** | **Present**   - The clinical problem - The hypothesis - A statement of objectives that includes the study population, the condition of interest, the exposure or intervention, and the outcome(s) considered | - **3** - **4** - **4** |
| **Sources** | **Describe**   - - Qualifications of searchers (e.g. librarians and investigators)   - Search strategy, including time period included in the synthesis and keywords   - Effort to include all available studies, including contact with authors   - Databases and registries searched   - Search software used, name and version, including special features used (e.g. explosion)   - Use of hand searching (e.g. reference lists of obtained articles)   - List of citations located and those excluded, including justification   - Method of addressing articles published in languages other than English   - Method of handling abstracts and unpublished studies   - Description of any contact with authors | - - **5**   - **5**   - **5**   - **5**   - **5**   - **5**   - **8/9**   - **6**   - **6**   - **n/a** |
| **Study Selection** | **Describe**   - Types of study designs considered - Relevance or appropriateness of studies gathered for assessing the hypothesis to be tested - Rationale for the selection and coding of data (e.g. sound clinical principles or convenience) - Documentation of how data were classified and coded (e.g. multiple raters, blinding and interrater reliability) - Assessment of confounding (e.g. comparability of cases and controls in studies where appropriate) - Assessment of study quality, including blinding of quality assessors; stratification or regression on possible predictors of study results - Assessment of heterogeneity - Statistical methods (e.g. complete description of fixed or random effects models, justification of whether the chosen models account for predictors of study results, dose-response models, or cumulative meta-analysis) in sufficient detail to be replicated) | - **5** - **5** - **5** - **5** - **7** - **7** - **7** - **7/8** |
| **Results** | **Present**   - A graph summarizing individual study estimates and the overall estimate - A table giving descriptive information for each included study - Results of sensitivity testing (eg, subgroup analysis) - Indication of statistical uncertainty of findings | - **26** - **23/4** - **26** - **11** |
| **Discussion** | **Discuss**   - Strengths and weaknesses - Potential biases in the review process (eg, publication bias) - Justification for exclusion (eg, exclusion of non–English-language citations) - Assessment of quality of included studies - Consideration of alternative explanations for observed results - Generalization of the conclusions (ie, appropriate for the data presented and within the domain of the literature review) - Guidelines for future research - Disclosure of funding source | - **12-13/15-16** - **13** - **16** - **11** - **13/14** - **17** - **14-17** - **-** |

| **eTable 2:** PRISMA guidelines for meta-analysis and systematic reviews | | | |
| --- | --- | --- | --- |
| **Section/topic** |   **#** | **Checklist item** |      **Page** |
| **TITLE** | | |  |
| Title |   1 | Identify the report as a systematic review, meta-analysis, or both. | 1 |
| **ABSTRACT** | | |  |
| Structured summary | 2 | Provide a structured summary including, as applicable: background; objectives; data sources; study eligibility criteria, participants, and interventions; study appraisal and synthesis methods; results; limitations; conclusions and implications of key findings; systematic review registration number. | 2 |
| **INTRODUCTION** | | |  |
| Rationale | 3 | Describe the rationale for the review in the context of what is already known. | 3 |
| Objectives | 4 | Provide an explicit statement of questions being addressed with reference to participants, interventions, comparisons, outcomes, and study design (PICOS). | 4 |
| **METHODS** | | |  |
| Protocol and registration |   5 | Indicate if a review protocol exists, if and where it can be accessed (e.g., Web address), and, if available, provide registration information including registration number. | 4 |
| Eligibility criteria | 6 | Specify study characteristics (e.g., PICOS, length of follow-up) and report characteristics (e.g., years considered, language, publication status) used as criteria for eligibility, giving rationale. | 5 |
| Information sources | 7 | Describe all information sources (e.g., databases with dates of coverage, contact with study authors to identify additional studies) in the search and date last searched. | 5 |
| Search | 8 | Present full electronic search strategy for at least one database, including any limits used, such that it could be repeated. | 5 |
| Study selection | 9 | State the process for selecting studies (i.e., screening, eligibility, included in systematic review, and, if applicable, included in the meta-analysis). | 5-6 |
| Data collection process | 10 | Describe method of data extraction from reports (e.g., piloted forms, independently, in duplicate) and any processes for obtaining and confirming data from investigators. | 6 |
| Data items | 11 | List and define all variables for which data were sought (e.g., PICOS, funding sources) and any assumptions and simplifications made. | 6 |
| Risk of bias in individual studies | 12 | Describe methods used for assessing risk of bias of individual studies (including specification of whether this was done at the study or outcome level), and how this information is to be used in any data synthesis. | 7 |
| Summary measures | 13 | State the principal summary measures (e.g., risk ratio, difference in means). | 7 |
| Synthesis of results | 14 | Describe the methods of handling data and combining results of studies, if done, including measures of consistency (e.g., I^2^) for each meta-analysis. | 7-8 |
| Risk of bias across studies | 15 | Specify any assessment of risk of bias that may affect the cumulative evidence (e.g., publication bias, selective reporting within studies). | 8 |
| Additional analyses | 16 | Describe methods of additional analyses (e.g., sensitivity or subgroup analyses, meta-regression), if done, indicating which were pre-specified. | 8 |
| **METHODS** |  |  |  |
| Study selection | 17 | Give numbers of studies screened, assessed for eligibility, and included in the review, with reasons for exclusions at each stage, ideally with a flow diagram. | 25 |
| Study characteristics | 18 | For each study, present characteristics for which data were extracted (e.g., study size, PICOS, follow-up period) and provide the citations. | 23-24 |
| Risk of bias within studies | 19 | Present data on risk of bias of each study and, if available, any outcome level assessment (see item 12). | 11 |
| Results of individual studies | 20 | For all outcomes considered (benefits or harms), present, for each study: (a) simple summary data for each intervention group (b) effect estimates and confidence intervals, ideally with a forest plot. | 26 |
| Synthesis of results | 21 | Present results of each meta-analysis done, including confidence intervals and measures of consistency. | 26 |
| Risk of bias across studies | 22 | Present results of any assessment of risk of bias across studies (see Item 15). | 12 |
| Additional analysis | 23 | Give results of additional analyses, if done (e.g., sensitivity or subgroup analyses, meta-regression [see Item 16]). | 11-12 |
| **DISCUSSION** |  |  |  |
| Summary of evidence |   24 | Summarize the main findings including the strength of evidence for each main outcome; consider their relevance to key groups (e.g., healthcare providers, users, and policy makers). | 12 |
| Limitations | 25 | Discuss limitations at study and outcome level (e.g., risk of bias), and at review-level (e.g., incomplete retrieval of identified research, reporting bias). | 13 |
| Conclusions | 26 | Provide a general interpretation of the results in the context of other evidence, and implications for future research. | 14-17 |
| **FUNDING** |  |  |  |
| Funding | 27 | Describe sources of funding for the systematic review and other support (e.g., supply of data); role of funders for the systematic review. | - |

| **eTable 3**: Features of each intervention strategy | |
| --- | --- |
| **Initiative** | **Description of intervention** |
| **CIEIS^29^** | **Community Interventions**:   - Workshop to increase awareness of early psychosis symptoms - Refresher session - Link to each community organisation - Educational and promotional materials - More direct referral pathway |
| **DETECT^25^** | **Healthcare Professional Training**:   - Presentations at conferences and education sessions for GPs - Articles in GP newspapers and journals - Newsletters and information sheets   **Community Interventions**:   - Articles in university newspapers - National mental health awareness campaign - Information website |
| **EASY/JCEP^26^** | **Community Interventions**:   - Public talks and exhibitions - More direct referral pathway - Public education campaigns - New Chinese term for psychosis |
| **EDEN^28^** | **Community Interventions**:   - Advertisements in newspapers, magazines and online - Posters and leaflets - Psychosis information line - Information website - Public talks and exhibitions |
| **EPIP^17^** | **Community Interventions**:   - Public talks and exhibitions - Newspaper advertising - Radio advertising - Postcard advertising - Posters - Information website - Information phone line   **Healthcare Professional Training**:   - Information newsletter - Talks, forums and workshops - Consultation restructuring |
| **EPPIC^18^** | **Standalone FEP service** |
| **EPPIC^24^** | **Community Interventions**   - Visits to secondary schools   **Healthcare Professional Training:**   - Talks, forums and workshops - Information newsletter - Information video |
| **IMAGES^30^** | **Healthcare Professional Training:**   - Talks, forums and workshops |
| **LEOCAT^21^** | **Healthcare Professional Training:**   - Information video - Talks, forums and workshops - Information leaflets - Refresher sessions - Case-by-case discussions |
| **OASIS^20^** | **Standalone CHR service** |
| **PAE-TPI^46^** | **Standalone FEP service** |
| **PEPP^50^** | **Community Intervention:**   - Posters - Leaflets and paper advertisements - Information video - Talks, forums and workshops   **Healthcare Professional Training:**   - Information leaflets - Follow up phone calls |
| **PEPP^27^** | **Healthcare Professional Training:**   - Information videos - Case discussions |
| **REDIRECT^51^** | **Healthcare Professional Training:**   - Information videos - Case discussions - Refresher sessions |
| **STEP^45^** | **Community Interventions:**   - Social media campaign - Radio advertising - Television advertising - Information website   **Healthcare Professional Training:**   - Information leaflets - Online resources - Talks, forums and workshops - Referral sources |
| **TIPS^15^** | **Community Interventions:**   - Newspaper advertising - Radio advertising - Television advertising   **Healthcare Professional Training**   - Targeted information campaign |

| **eTable 4: Inclusion/exclusion criteria across the studies included in the meta-analysis** | |
| --- | --- |
| **Initiative** | **Inclusion/Exclusion Criteria** |
| **CIEIS^29^** | **Inclusion**: All individuals referred to service with a DSM-IV defined FEP  **Exclusion:** N/A |
| **DETECT^25^** | **Inclusion**: All individuals referred to service with a DSM-IV defined FEP  **Exclusion:** N/A |
| **EASY/JCEP^26^** | **Inclusion**: Cantonese-speaking Chinese, FEP with a diagnosis of schizophrenia, schizophreniform disorder, brief psychotic disorder, delusional disorder or psychosis not otherwise specified according to DSM-IV criteria.  **Exclusion**: Subjects with affective psychotic disorders and psychosis related to substance abuse; having significant neurological disorders and intellectual disability. |
| **EDEN^28^** | **Inclusion**: All individuals referred to service with a DSM-IV defined FEP  **Exclusion:** N/A |
| **EPIP^17^** | **Inclusion:** All individuals referred to service with a DSM-III-R defined FEP  **Exclusion:** Previous psychiatric consultation and  those where a physical cause was found for psychosis, drug-induced psychosis |
| **EPPIC^18^** | **Inclusion**: Age at onset for first psychotic episode between 16 and 30 and currently psychotic according to DSM-III-R criteria.  **Exclusion:** Intellectual disability, a clear organic cause for psychosis, epilepsy, and inadequate command of English. |
| **EPPIC^24^** | **Inclusion**: Aged 16–30, experiencing a first psychotic episode (defined by DSM-IV criteria), have no prior history of treatment for a psychotic illness, be fluent in English, and have stable accommodation within the EPPIC catchment area.  **Exclusion**: Intellectual disability or a clear organic cause for psychosis. Given that the study aimed to assess the impact of a community intervention, clients without permanent residence in the area were also excluded. |
| **IMAGES^30^** | **Inclusion**: Aged 18-65, diagnosis of DSM-IV schizophrenia, absence of comorbid neurological or psychiatric disorders or substance abuse, and ability to provide informed consent  **Exclusion:** N/A |
| **LEOCAT^21^** | **Inclusion**: Aged 16–35, presenting to local mental health services for the first time with DSM-IV defined FEP.  **Exclusion**: Not their first treated episode or presentation, i.e. they had a history of contact with mental health services for psychosis for more than 6 months or antipsychotic treatment for more than a month (with greater than 50% treatment adherence). |
| **OASIS^20^** | **Inclusion:** Intervention group only – individuals referred to service with a DSM-IV defined FEP previously recruited to OASIS and met PACE (personal assessment and crisis evaluation) criteria for an at risk mental state (ARMS) for psychosis**.** Control group only -individuals referred to service with a DSM-IV defined FEP never having been recruited to OASIS  **Exclusion:** Control only – patients who had previously contacted mental health services with prodromal symptoms |
| **PAE-TPI^46^** | **Inclusion**: All individuals referred to service with a DSM-IV defined FEP  **Exclusion:** N/A |
| **PEPP^50^** | **Inclusion**: Aged 14–30; meeting DSM-IV criteria for a psychotic disorder not attributed to an organic brain condition or clearly substance induced; not having received antipsychotic medication for a period 1 month before referral; and an IQ above 70  **Exclusion:** N/A |
| **PEPP^27^** | **Inclusion**: Aged 14–30; meeting DSM-IV criteria for a psychotic disorder not attributed to an organic brain condition or clearly substance induced; not having received antipsychotic medication for a period 1 month before referral; and an IQ above 70  **Exclusion:** N/A |
| **REDIRECT^51^** | **Inclusion**: Aged 14-30, consecutive patients with ICD-10 defined FEP referred to early-intervention services.  **Exclusion**: Patients with a primary diagnosis of substance use disorder, mood disorder, or organic mental disorder, current criminal proceedings, serious concurrent physical illness, institutional residence, learning disability, or inability to provide informed consent. |
| **STEP^45^** | **Inclusion:** Aged 16–35, had their DSM-IV defined FEP psychosis within the past 3 years.  **Exclusion:** Established diagnosis of affective psychosis (i.e. non-ambiguous bipolar or MDD with psychotic features) or psychosis secondary to substance use or a medical illness, unable to communicate in English, eligible for DDS (Department of Developmental Services), legally mandated to enter treatment, unable to reliably determine DUP, unstable serious medical illness; patients who converted to psychosis while being followed and cared for in prodromal clinics (i.e. DUP of 0), which exist at both sites; previously received care at another early intervention service |
| **TIPS^15^** | **Inclusion**: Aged 18-65; meeting the DSM-IV criteria for schizophrenia, schizophreniform disorder, schizoaffective disorder (narrow schizophrenia spectrum disorders) or brief psychotic episode, delusional disorder, affective psychosis with mood-incongruent delusions, or psychotic disorder not otherwise specified; being actively psychotic, as measured by a Positive and Negative Syndrome Scale (PANSS); not receiving previous adequate treatment for psychosis; having no neurological or endocrine disorders with relationship to the psychosis; having no contraindications to antipsychotic medication; understanding and/or speaking a Scandinavian language; having an IQ score of above 70; and being willing and able to give informed consent  **Exclusion:** N/A |

***eMethods1***

Evaluation of study quality on all included studies was performed using a risk bias tool, an adapted version of the Newcastle Ottawa Scale^28^. Information was collected on representativeness of intervention and control groups, ascertainment of exposure, demonstration of outcome of interest not present at start of study, comparability of cohorts, assessment of outcome, length of study, and completeness of data.

**eFigure 1**: Meta-regression analyses. Effect of controlled intervention on the DUP (Hedge’s g) by age

**eFigure 2**: Meta-regression analyses. Effect of controlled intervention on the DUP (Hedge’s g) by gender (% male)

**eFigure 3**: Meta-regression analyses. Effect of controlled intervention on the DUP (Hedge’s g) by study quality

**eFigure 4**: Meta-regression analyses. Effect of controlled intervention on the DUP (Hedge’s g) by publication year

**eFigure 5**: Meta-regression analyses. Effect of controlled intervention on the DUP (Hedge’s g) by percentage of married subjects

**eFigure 6**: Meta-regression analyses. Effect of controlled intervention on the DUP (Hedge’s g) by study design

**eFigure 7**: Meta-regression analyses. Effect of controlled intervention on the DUP (Hedge’s g) by DUP endpoint definition

**eFigure 8**: Meta-regression analyses. Effect of controlled intervention on the DUP (Hedge’s g) by continent where study took place

**eFigure 9**: Meta-regression analyses. Effect of controlled intervention on the DUP (Hedge’s g) by healthcare system type

**eFigure 10**: Funnel plot of standard error by Hedge’s g

**eFigure 11**: Funnel plot of precision by Hedge’s g

**eFigure 12**: Sensitivity analysis displaying summary meta-analytical effect with each study removed each time
